# Arthrodesis and Defect Bridging of the Upper Ankle Joint with Allograft Bone Chips and Allograft Cortical Bone Screws (Shark Screw^®^) after Removal of the Salto-Prosthesis in a Multimorbidity Patient: A Case Report

**DOI:** 10.3390/life12071028

**Published:** 2022-07-11

**Authors:** Klaus Pastl, Eva Pastl, Daniel Flöry, Gudrun H. Borchert, Michel Chraim

**Affiliations:** 1 Department for Orthopedic Surgery Diakonissenhospital Linz, Weißenwolfstr. 15, 4020 Linz, Austria; klaus@pastl.at (K.P.); eva@pastl.at (E.P.); daniel.floery@diakonissen.at (D.F.); 2 Dr. Borchert Medical Information Management, Egelsbacher Str. 39e, D-63225 Langen, Germany; 3 Fusszentrum Wien, Alser Straße 43/Top 8d, 1080 Vienna, Austria; chraim.michel@gmail.com

**Keywords:** arthrodesis, upper ankle joint, allograft, multimorbidity, cortical bone screw (Shark Screw^®^), case report

## Abstract

The case describes the revision of an upper ankle prosthesis because of loosening. When ankle replacement is the first choice and actual bone quality does not allow a replacement of the prosthesis, arthrodesis is the only way of reducing pain and gaining stability. The amount of missing bone due to the removed prosthesis was severe. Shark Screws^®^, made of human allograft cortical bone, were used to fix an allograft femoral head and tibia as well as fibula and talus to each other for stabilization. This was performed without any autologous bone graft and without metal screws. The human matrix of the cortical allograft allows the creation of new vessels followed by osteoblastic activity and production of new bone. The revascularization of the allografts reduces the risk of infection and wound problems. Over time, the patient’s bone metabolism allows the allografts to be remodeled into the patient’s bone. The case reported here had severe multimorbidity. The loosening of the prosthesis mainly affected the ability to perform housework, mobility, enjoying leisure, and it had a great impact on the emotion and well-being of the patient. One year after surgery, the patient is very satisfied to be able to walk without pain and scratches for about 90 min.

## 1. Introduction

When ankle replacement is the first choice, and bone quality does not allow a replacement of the prosthesis, arthrodesis is the only way of reducing pain and gaining stability. The case reported here describes the revision of an upper ankle prosthesis because of loosening. The patient had a Salto-prosthesis for 14 years and now, at the age of 78 years, an arthrodesis was performed. The amount of missing bone due to the removed prosthesis was severe. The amount of allograft used was therefore very high. Shark Screws^®^, made of allograft cortical bone, were used to fix an allograft femoral head and tibia, fibula and talus to each other for stabilization. This was performed without any autologous bone graft and without metal screws.

**Figure 1:**
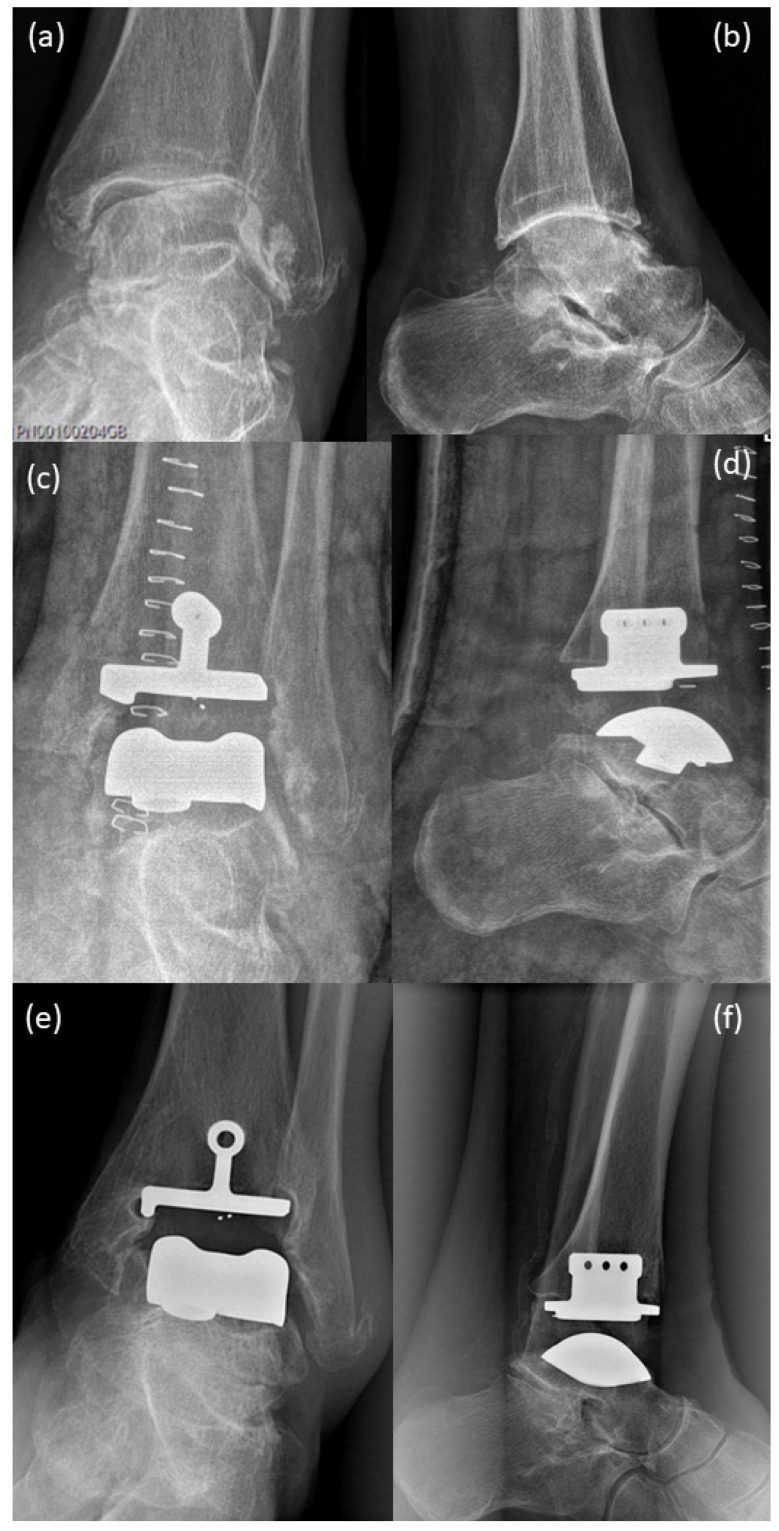
X-rays: (**a**,**b**) before implantation of Salto-prosthesis, pre-OP 2005; X-rays show severe osteoarthrosis of the upper ankle with deformity of joint endplates, osteophyte formation and subchondral sclerosis, as well as periarticular calcifications; (**c**,**d**) post-surgical 2006, implanted Salto-prosthesis; with regular post-surgical status; (**e**,**f**) X-ray at a routine control performed in 2016 demonstrated beginning of bone resorption and lytic–cystic defects in the periphery of the tibial component while still regularly around the stem of the prosthesis and the talar component.

**Figure 2:**
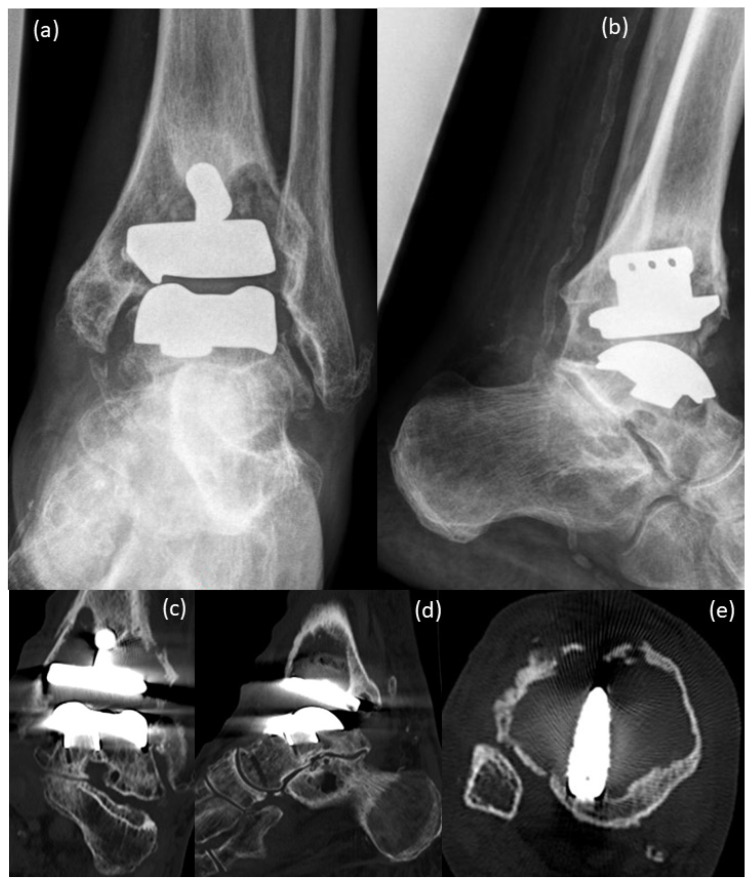
Pre-surgical images 2020: (**a**,**b**) X-rays, (**c**–**e**) CT scans of the patient; (**a**,**b**) large cysts are present in the tibia and in the talus, compared to the images from the year 2016 (Figure 1), a massive increase in bone resorption and cyst formation is seen around the tibial component under involvement of the stem of the prosthesis. Therefore, an increasing tilt of the joint surface of the tibial component can be seen as a sign of incipient dislocation; (**c**–**e**) CT scans confirm the X-ray findings with massive bone resorption, pathologic fracture of the lateral tibial cortical surface and instability of the tibial prosthesis component.

For decades, ankle fusion has been the first choice in the management of end-stage ankle osteoarthritis [1,2]. However, total ankle replacement has evolved to be a viable alternative [1,2]. Especially in younger patients, this is one of the options due to the range of motion, and therefore, the quality of life is higher after total ankle arthroplasty [2]. However, the survivorship is lagging behind that of hip and knee replacements (5 years in 87% of the patients [1] and 81% after 7 years [3] for Salto-prosthesis), with failure rates for replaced ankles approximately double (19% [3]) compared with hips and knees [1]. It is still debated whether ankle replacement or ankle fusion is better [2,4]. Neither total ankle replacement nor arthrodesis restored normal movement and walking speed [5]. Haddad et al. [6] could not find any difference in the outcome between patients with ankle arthroplasty and ankle arthrodesis. In their meta-analysis, they obtained a survival rate of the implant of 77% after 10 years [6]. The prosthesis of the case reported here survived 14 years, which was a good intermediate step, but strong osteolysis, already seen in 2016 (Figure 1e,f), dramatically increased by 2020 (Figure 2), requiring arthrodesis to treat this condition. Ankle arthrodesis is still the primary treatment for end-stage osteoarthritis of the ankle joint, with a 9% failure rate and 22% postoperative complications. An estimated 6% of patients needed a re-operation due to irritation of the hardware [7]. Successful fusion requires bony coaptation, compression and rigid immobilization [8]. This is obtained mostly with open joint debridement and crossed lag-screw fixation; however, when the above three principles are followed, successful outcomes have been reported with a variety of fixation devices [8]. The method of fixation presented here did not use crossed lag-metal screws but used several allograft cortical bone screws and other allografts for stabilization. Infection is one of the main complications when performing total ankle arthroplasty [9] or arthrodesis. Using allograft cortical bone screws for arthrodesis reduces the risk of infection due to the naturally occurring bone metabolism [10], and there is no irritation due to hardware. Bone is built up where there is bone loss. Remodeling into the patient’s own bone always enables greater stability, and no hardware removal is necessary. The bigger the amount of allograft, the longer it takes to transform the allograft into vital bone [11]. In the case reported here, there were no signs of rejection. After 10 weeks, Brcic et al. [10] could show that blood vessels and all bone cells were present in the allograft cortical bone screw, and it resulted in fast primary bone healing without immunological rejection. Cortical allografts heal first by allograft osteoclastic resorption and creation of new vessels, followed by osteoblastic activity and production of new bone [12]. The incorporation of a cancellous bone allograft is a process called “creeping substitution”, in which the grafted bone is simultaneously resorbed and replaced by the newly formed bone from the host bone [13].

The aim of this surgical procedure was to regain stability and enable the patient to walk without scratches. Using only allografts for the arthrodesis of an upper ankle after removing a prosthesis is unique. Even for the osteosynthesis, allogeneic cortical bone screws were used. All allografts ultimately lead to a stable bony connection. No metal remains but the patient’s own bone. Even with the high number of comorbidities, the arthrodesis was successful and strongly improved the quality of life of the patient. To our knowledge, no arthrodesis for such condition has been reported up to now.

## 2. Case Presentation

The patient was a hotel and restaurant owner who was standing most of his working day/life on hard floor (tiles) and lifting heavy weights several hours a day. He was a heavy smoker for over 25 years, with up to 40 cigarettes per day, and was passive smoking due to the work in the restaurant. There are no foot pathologies known in the family. The patient had the surgery, despite a very high grade of multimorbidity (Table 1).

Because of an old ligament tear on the left upper ankle (1970) and because of severe pain since 1992 under nonsteroidal anti-inflammatory drugs daily, his walking distance was max. 1 h. The X-ray images show severe osteoarthrosis of the upper ankle with deformity of the joint endplates, osteophyte formation and subchondral sclerosis, as well as periarticular calcifications (Figure 1a,b, 2005). Dorsiflexion of the left ankle was 20-0-20 (2006), lateral ligament stable, marked crepitation and marked drawer in the upper ankle joint, pronounced swelling. In 2006, he underwent surgery to implant an upper ankle joint prosthesis. Post-surgery images (2006, Figure 1c,d) show implanted Salto-prosthesis (Tornier GmbH, Burscheid, Germany) with regular post-surgical status. The prosthesis was a first-generation prosthesis (two components). This first-generation prosthesis showed disappointing results over the long term [14].

X-ray of a routine control performed in 2016 (Figure 1e,f) demonstrated the beginning of bone resorption and lytic–cystic defects in the periphery of the tibial component while still regularly around the stem of the prosthesis and the talar component. The patient already suffered pain at this time.

In July 2020, the patient returned after a fall 2 weeks before, where he injured the left ankle. An erysipel was diagnosed and treated in another hospital and was treated with antibiotics, but the pain did not decrease, and an X-ray was taken. Clinically, the left ankle joint was clearly swollen, but mobility was still maintained. Any load on the foot was painful. The patient walked with two crutches. For immobilization the patient obtained a Vacuped (OPED AG, Steinhausen, Switzerland) shoe until surgery. The pain and swelling were still increasing. The patient attended an extended clarification interview with exact surgery planning and no weight bearing on the left foot due to the severe bone defects until surgery (November 2020). The AOFAS score at the time of surgery was 7.

The X-ray images from 2020, pre-surgery, showed large cysts in the tibia and in the talus (Figure 2a,b). Compared to the images of the year 2016, a massive increase in bone resorption and cyst formation was seen around the tibial component under involvement of the stem of the prosthesis. Consequently, an increasing tilt of the joint surface of the tibial component could be seen as a sign of incipient dislocation (Figure 2a,b). CT scans before surgery (Figure 2c–e) confirmed the X-ray findings with massive bone resorption, pathologic fracture of the lateral tibial cortical surface and instability of the tibial prosthesis component.

## 3. Surgical Procedure

**Figure 3:**
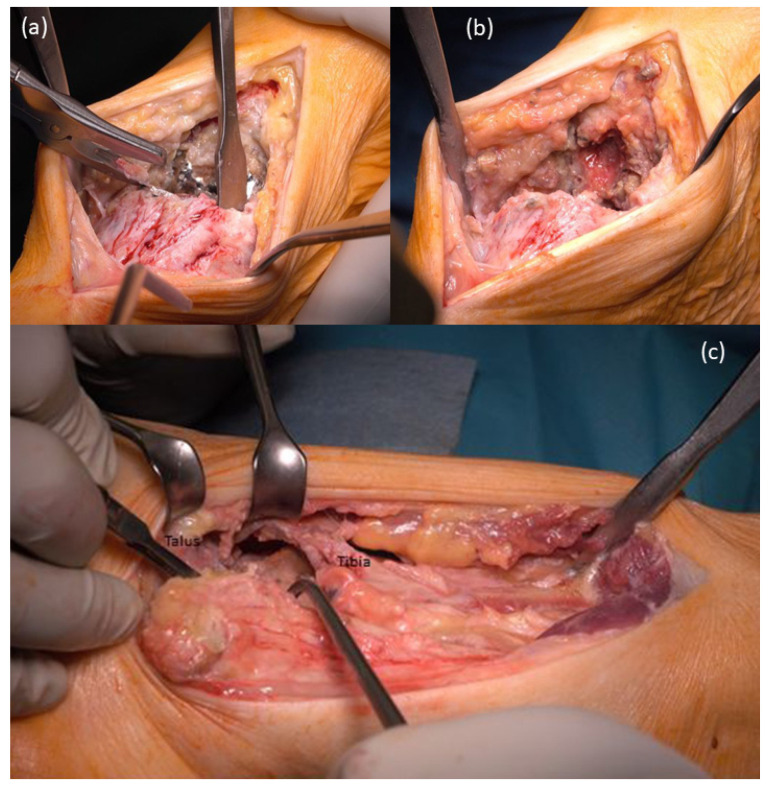
Surgical procedure: (**a**) Salto-prosthesis and necrotic talus; (**b**) after removal of the Salto-prosthesis bony defects and metal abrasion is visible; (**c**) bony defects on talus and tibia with metal abrasion.

In the supine position and under application of a tourniquet cuff, a straight incision along the tibialis anterior tendon was performed with direct access to the prosthesis (Figure 3a); removal of the loose prosthesis was performed, and a careful synovectomy was performed to clean and refresh the vital bone areas. The talus was only preserved as a 1 cm thick bone clod and showed large cavities. The distal part of the tibia also showed large osteolysis and only a thin-walled cortex. Additional lateral approach along the fibula. Intra-articular massive synovitis and metal abrasion were visible (Figure 3b,c).

**Figure 4:**
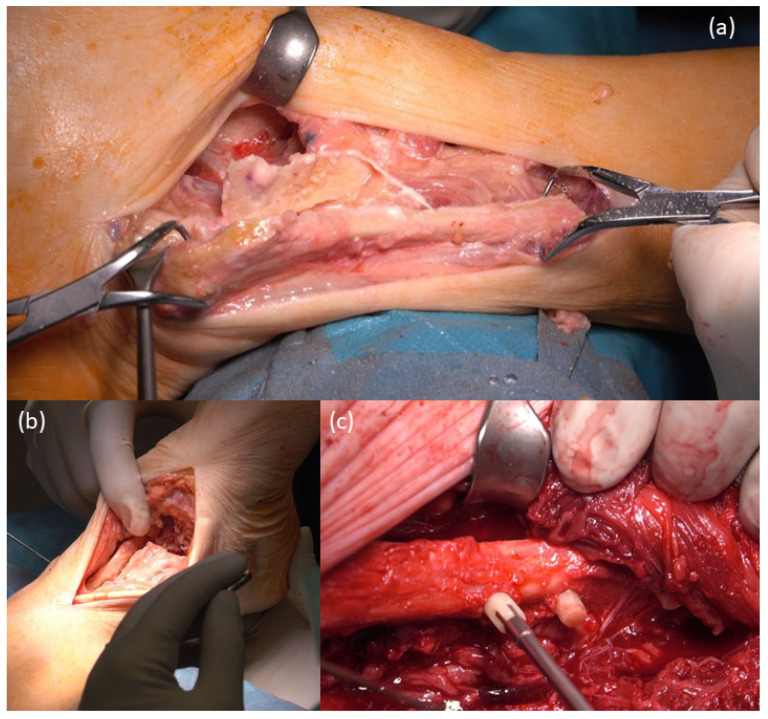
Preparation of the fibula: (**a**,**b**) Shifting the split and pedicled fibula downward; (**c**) The fibula was fixed with a Shark Screw^®^ diver (left screw) and with Shark Screws^®^ cut (all 5 mm in diameter) toward the tibia.

The fibula was cut 7 cm proximal to the joint space. In addition, the fibula was split longitudinally, mobilized and displaced, so that the spongy surface of the fibula could be fixed with Shark Screws^®^, 5 mm in diameter, to the tibia and talus (Figure 4a,b). The fibula was fixed toward the tibia from the lateral side with one Shark Screw^®^ diver and three Shark Screws^®^ cut (all 5 mm in diameter, Figure 4c).

**Figure 5:**
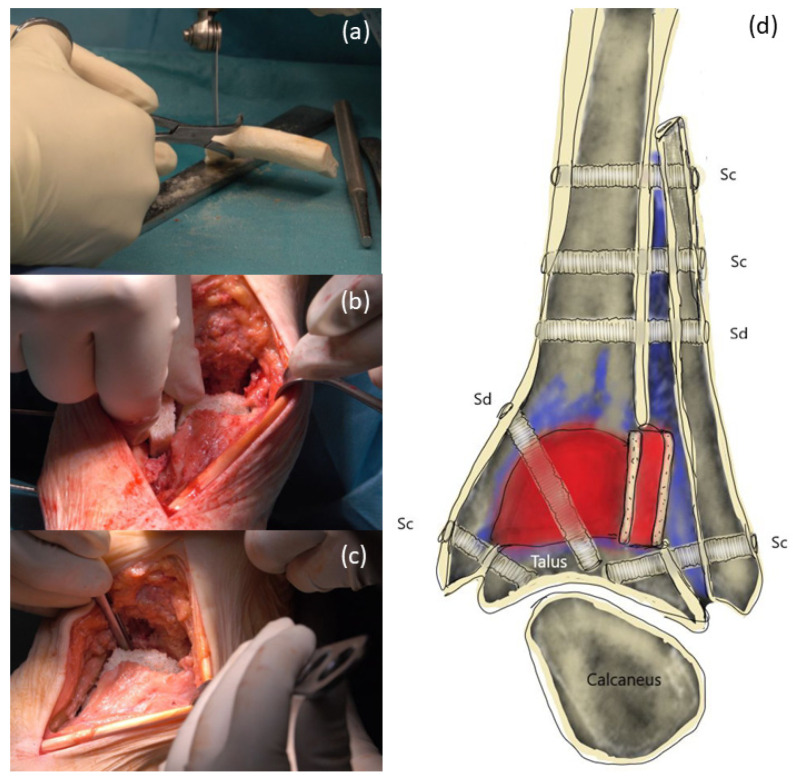
Filling the bony defects: (**a**) Preparation of the tricortical chip, for supporting the distal cortical tibia. (**b**) Insertion of the tricortical chip under the distal lateral tibial area, as an additional load-bearing column. (**c**) One femoral head was shaped and was inserted into the tibia. (**d**) Drawing of the procedure performed: red: inserted allograft, violet: empty cavities, later filled with demineralized bone matrix, Sc: Shark Screw^®^ cut, Sd: Shark Screw^®^ diver.

Preparation of the tricortical chip (Figure 5a). For further support, the tricortical chip was notched under the missing lateral cortical tibial area to create a bony column and connection (Figure 5b). A shaped femoral head (DIZG gGmbH, Berlin, Germany) was inserted into the tibia (Figure 5c). The remaining parts from shaping the femoral head were used to fill the cavities of the talus.

**Figure 6:**
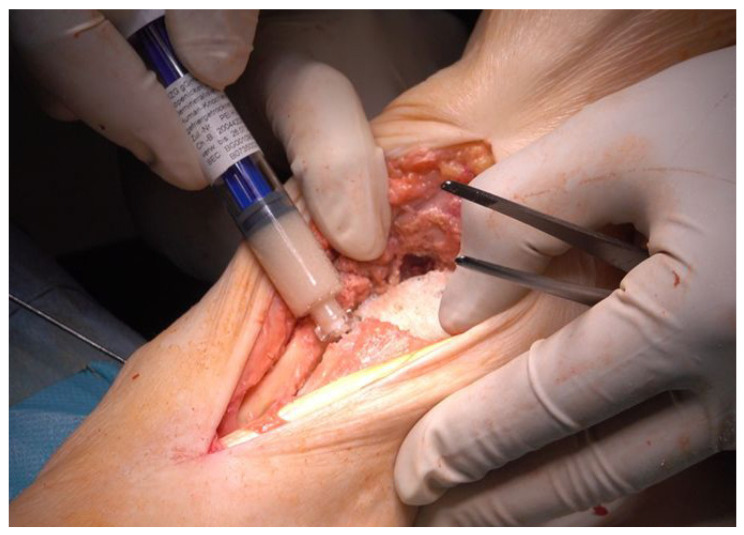
Filling the defects: Filling the gap between fibula and tibia and smaller defects with demineralized bone matrix (DBM), inserted femoral head clearly visible.

All crevices and small cavities were filled with demineralized bone matrix (Figure 6, DIZG gGmbH, Berlin, Germany).

**Figure 7:**
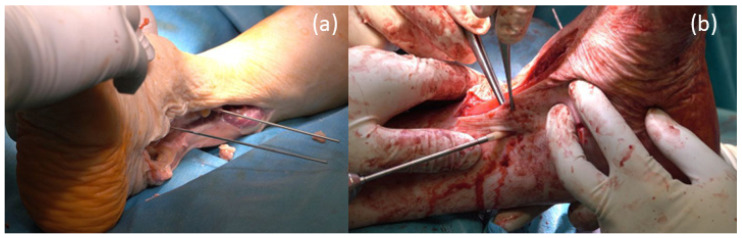
Arthrodesis of the upper ankle joint: (**a**) Temporary placement of the drill wires and fixation of the fibula to the tibia; (**b**) Placement of a Shark Screw^®^ diver from the tibia through the inserted femoral head for fixation of the talus.

The osteosynthesis between tibia and fibular was performed with 2 Shark Screws^®^ cut 3.5 cm long and 5 mm in diameter and with one Shark Screw^®^ diver 3.5 cm long and 5 mm in diameter. Temporary fixation of the fibula to the tibia was obtained with k-wires (Figure 7a). The lower connection between the tibia and talus was performed with 1 Shark Screw^®^ diver 4.5 cm long, 5 mm diameter through the inserted femoral head, and 1 Shark Screw^®^ cut, 5 mm in diameter. Additional stabilization was obtained by placing 1 Shark Screw^®^ cut over the distal part of the fibula into the talus (Figure 7b).

Wound closure in layers was performed after an accurate hemostasis. In the end, a split plaster cast was applied on the lower leg, which was changed into a closed one after 5 days.

## 4. Postoperative Rehabilitation

Due to the condition of the patient, the physiotherapist came home in weeks 1–17 for rehabilitation:

Phase I: 

Week 1–7: bed exercises, lymphatic drainage, exercise with crutches and rollator, zero weight on the left foot.

Phase II: 

From week 7: Weight bearing with increasing weight, in-plaster walking exercises with partial weight bearing, lymphatic drainage, weight bearing from 5 kg (week 7) to 20 kg (week 9) on the leg, patient walks 10 m with crutches. Starting from week 10, weight bearing with half the body weight, short walk with crutches (15 m, limited by chronic obstructive pulmonary disease). Starting from week 11, full weight bearing, walking distance 2 × 15 m without break, balance training. Week 13: exchange of plaster to plaster with rolling cradle. Week 17: removal of plaster cast, walking with Vacoped shoe (OPED AG, Steinhausen, Switzerland).

Phase III: 

From week 18 onwards, therapy in the doctor’s office: lymphatic drainage, gait training with crutches, shoe fitting with roll-off function.

Phase IV: 

From week 20, patient walks freely without crutches.

## 5. Results

The patient reported pain only in the first week after surgery, in the hospital. Postoperative pain management: Infusion of Xefo 8 mg for 9 days, the first 2 days additional 1 g Paracetamol infusion. At discharge from hospital, if necessary, 8 mg Xefo in the morning and in the evening. Due to this pain management, there was no pain at discharge. Minor foot/skin pain for a few days in the phase of walking freely without crutches was noted too. He can now walk symptom free for about 90 min.

American Orthopaedic Foot and Ankle Society (AOFAS) score was obtained only before surgery and at the last follow-up. Before arthrodesis (October 2020), the AOFAS score was 7, and the part of the AOFAS Score for pain was “0”, thus, severe pain nearly all the time. At the last follow-up, 15 months after surgery, the AOFAS score increased to 79 (pain scored at 40 points, which is equivalent to no pain). The patient received a prescription for rolling gait shoes to further improve walking and the fluidity of motion.

**Figure 8:**
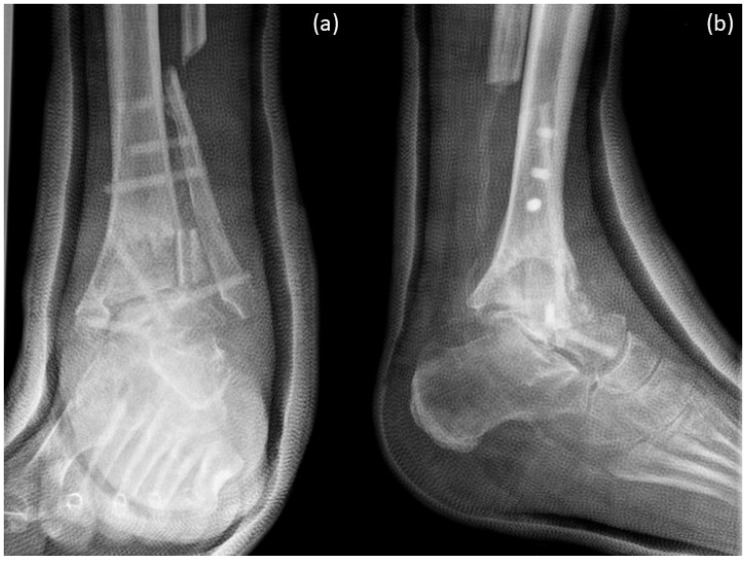
X-rays just after surgery. Arthrodesis was performed with 6 Shark Screws^®^ and a huge amount of allograft (femoral head and tricortical chip). (**a**) Ap-view; (**b**) lateral view.

X-rays were performed just after surgery, 12 weeks and 1 year after surgery. Figure 8 shows X-rays just after surgery. Arthrodesis was performed with 6 Shark Screws^®^ and a huge amount of allograft (femoral head and tricortical chip).

**Figure 9:**
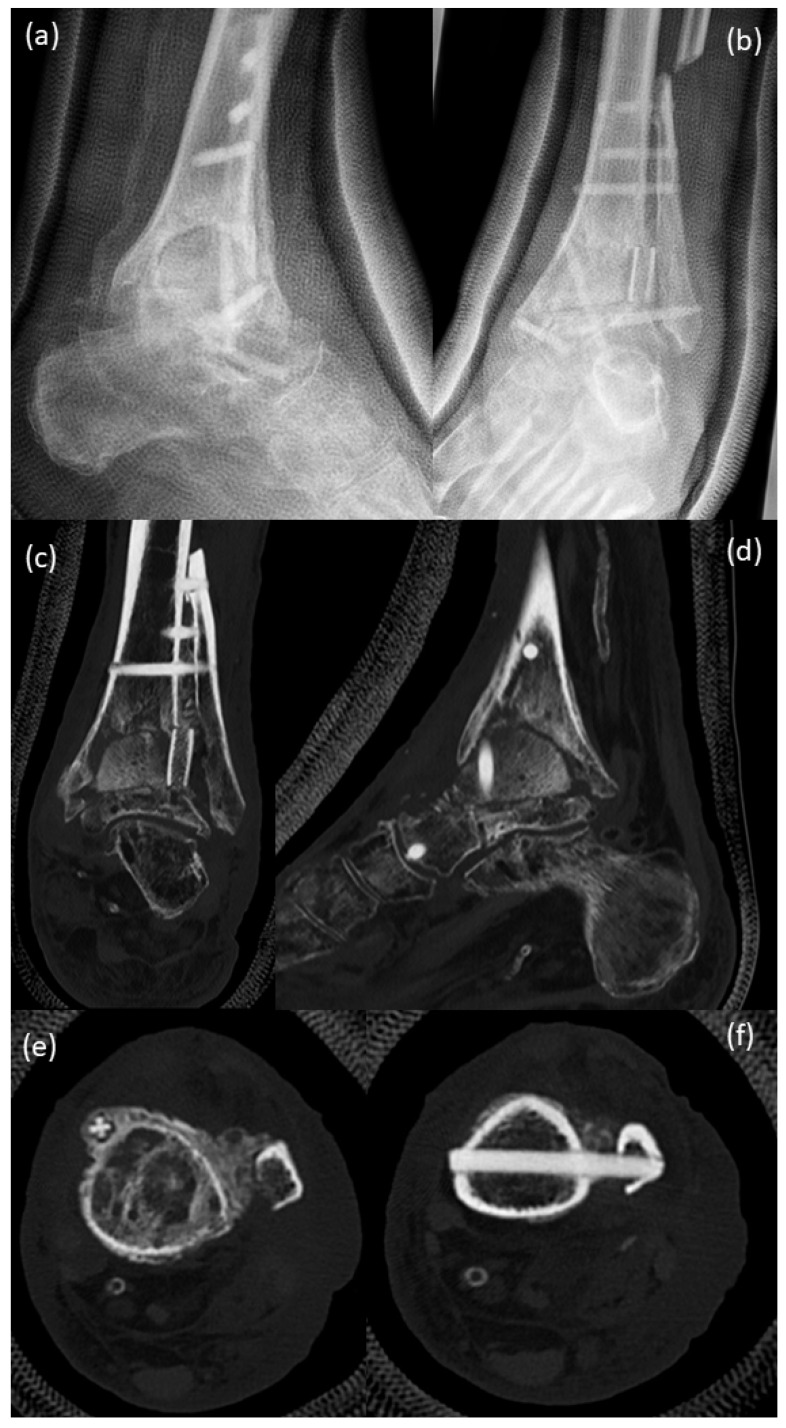
(**a**,**b**) X-ray; (**c**–**f**) CT scans 12 weeks after surgery with a clearly visible formation of callosal bone being seen around the screws with the beginning of bony consolidation.

Twelve weeks after surgery (Figure 9), a clearly visible formation of callosal bone was seen around the screws, with a beginning of bony consolidation. CT scans support the X-ray findings.

**Figure 10:**
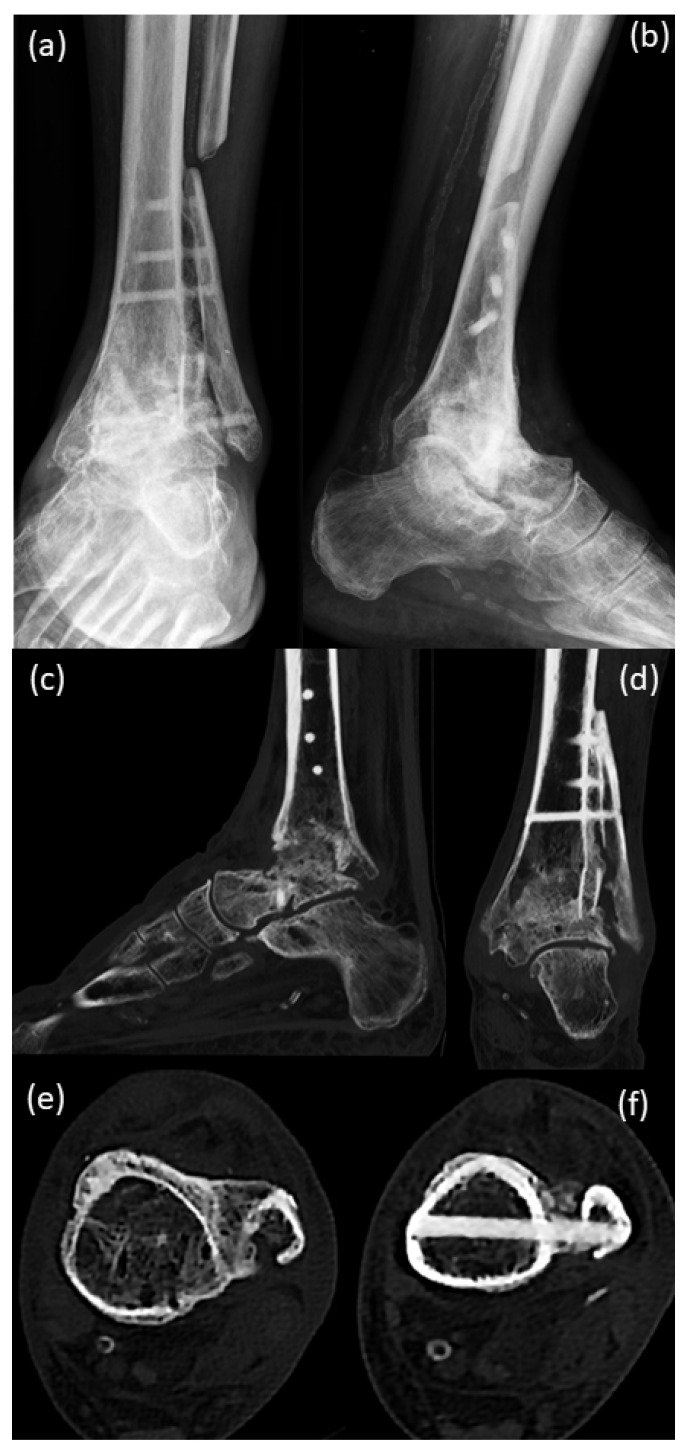
(**a**,**b**) X-rays; (**c**–**f**) CT scans 1 year after surgery. There is extensive callosal bone superstructing the distal fibular and tibia. It is clearly seen that, in particular, the situation within the distal tibia and the talus is in the status of good bony consolidation. No evidence of bone resorption or insufficiency of screws can be seen.

One year after surgery (Figure 10), there was an extensive callosal bone superstructing the distal fibular and tibia. It is clearly seen that, in particular, the situation within the distal tibia and the talus was now in the status of good bony consolidation. No evidence of bone resorption or insufficiency of screws was obvious.

**Figure 11:**
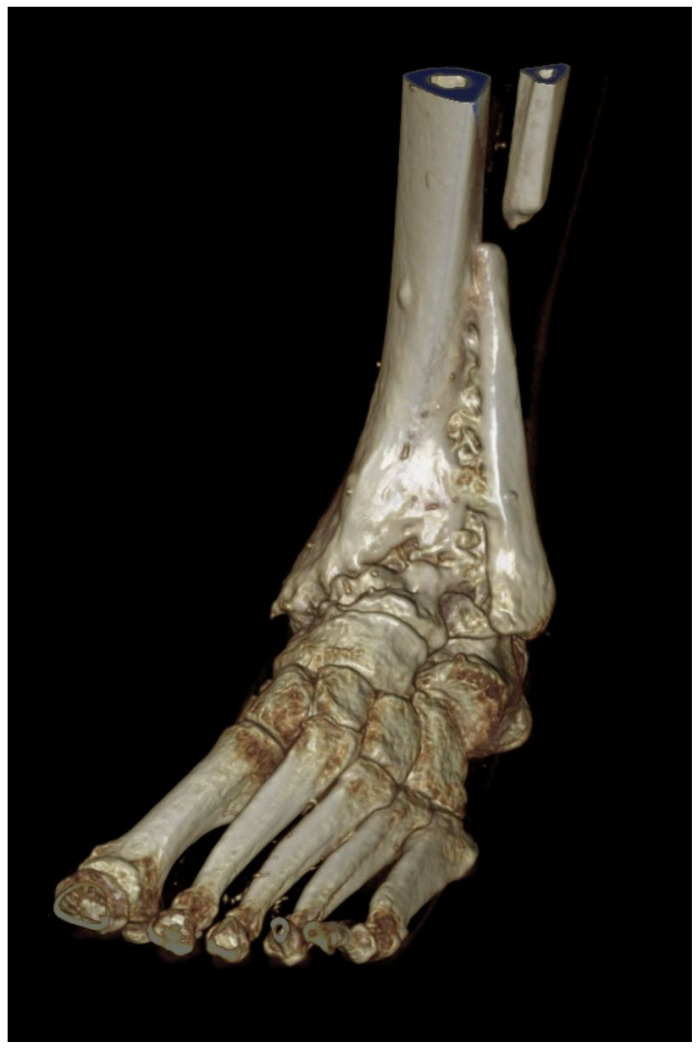
ROW-CT-3D-reconstruction 1 year after surgery. The arthrodesis of the fibula with the tibia is clearly visible.

A 3D scan was performed after 1 year (Figure 11). The arthrodesis of the fibula with the tibia is visible.

A well-consolidated arthrodesis due to the CT scan and 3D model was observed. High primary biomechanical stability was observed due to the use of Shark Screws^®^ in addition to allograft use (bone chips and femoral head). Allograft use avoided donor side morbidity and foreign material incorporation. The pain in the ankle joint before surgery was one of the reasons to ask for treatment. Before surgery, low (zero) points as part of the American Orthopaedic Foot and Ankle Society (AOFAS) score (severe pain nearly all the time) were recorded, which improved to full score (no pain at all) after surgery. Due to less pain, pain medication could be reduced (in a case of already massive existing medication intake).

There have been no adverse events recorded up to now.

## 6. Discussion

Surgical treatment of a revision of an ankle prosthesis is challenging [15]. Salvage arthrodesis after total ankle arthroplasty has a worse outcome than salvage of a primary fusion [2]. Even the new generation of ankle prostheses does not present a considerable improvement in survival [1,3]. Many difficulties are reported when arthrodesis of the upper ankle joint is performed [8]. This is due to various reasons, including (1) the fusion of a relatively small bone to a bone with a long lever; (2) relatively small cancellous bone surfaces for fusion; and (3) previous trauma, infection or damage caused by inflammatory arthritis with subsequent reduction in blood supply [8]. Further problems described by Muir [8] are poor soft tissue coverage in the ankle region caused by thin subcutaneous tissue, a lack of muscle coverage and poor venous return, contributing to early wound problems that have the potential of becoming deeply infected and permanently swollen [8]. If the possibility of total ankle arthroplasty was used in the past, arthrodesis is the only option. The patient described in this case report had massive bone resorption, and due to his very high multimorbidity, autologous bone harvest would not have been reasonable. Therefore, the only option was the use of allograft. An allograft femoral head and allograft chips were prepared to fill the big cavities, and demineralized bone matrix was used to fill the small cavities. Stabilization and compression of the allograft bone with the available bone stock was performed by using cortical bone screws (Shark Screw^®^). The patient had no early wound problems and no infections. This was probably due to the gentle treatment of the soft tissue when using the described procedure. The allografts used do support bone metabolism. Thus, no allergenic reaction was observed. Pseudarthrosis is often reported after ankle fusion [8]. The allograft cortical bone screws (Shark Screw^®^) used allow, additionally to the bone void filled with them, a tight connection of the bone at the arthrodesis side. The difference is that with the allograft cortical bone screw, the tight connection was achieved with bone and not with metal. This leads to a low number of pseudarthroses, as shown for other types of surgeries using this allograft cortical bone screw [16,17,18]. Several biomechanical studies described a higher torsional and bending strength when internal fixation systems were used in comparison to external fixation systems [19,20,21]. The Shark Screw^®^ is an internal fixation system. Additionally, Muir et al. [8] defined three principles for a successful arthrodesis: bony coaptation, compression and rigid immobilization [8]. All these principles were followed in the case reported, with tight coaptation and compression, using the allograft bone screws and seven weeks of full immobilization (no weight bearing). Long-term complications, such as malalignment, chronic edema and symptomatic hardware, were avoided using the described surgical technique. The post-surgical AOFAS score of 79 was similar to the value reported by Haddad et al. (AOFAS score: 76, [6]) in their review article, by Thomas et al. (AOFAS score: 74, [22]), by Buchhorn et al. (AOFAS score: 68–69, [23]) and Fischer et al. (AOFAS score: 53–55, [15]). Arthrodesis after removal of an upper ankle prosthesis with allograft bone and the allograft cortical bone screws is one option to regain activity. The alteration of the gait after arthrodesis should be addressed, as performed by our patient with specific shoe wear, but it is also described when arthroplasty is performed [5].

This case report shows in detail how to perform the arthrodesis of the upper ankle joint with huge bone defects after removal of a Salto-prosthesis. The arthrodesis was performed with allograft bone and allograft cortical bone screws without using any metal for fixation.

A limitation of the case report is that this is a single patient. Another limitation is that the prosthesis implanted was a first-generation prosthesis, and the technique of implantation had changed substantially by the present day, and the prosthesis may have survived longer with the knowledge of today. Performing more surgeries in patients with multimorbidity would support the finding and underline the described advantages. This case report has a follow-up of 15 months. Thus, degenerative changes of subjacent joints cannot be excluded.

## 7. Conclusions

The takeaway lessons of this case report are: (a) loosening of an upper ankle prosthesis with huge bone defects can be treated by using only allograft bone and allograft cortical bone screws for arthrodesis. No other materials were used. This leads to a high stability of the osteosynthesis. The revascularization of the allograft reduces the risk of infection and wound problems. Finally, it leads to an extensive callosal bone superstructing; (b) internal fixation allows high torsional and bending strength; (c) no harvest of autograft and no donor site morbidity; (d) no metal hardware and any removal necessary; (e) as reported for other techniques, an improvement of orthopedic scores, even in patients with very high co-morbidities, can be obtained using this “allogeneic arthrodesis”.

The decisive therapy goal here was to achieve a pain-free ankle joint that can bear weight and a plantigrade foot [24]. Patient perspective: The patient is very satisfied to walk without pain and scratches for about 90 min. The goal of the therapy was achieved.

## Figures and Tables

**Table 1:** Patient’s multimorbidity and medication.

Orthopedic Morbidities	Non-Orthopedic Morbidities	Medication
ligament rupture of left upper ankle joint, no operation, 1970	Diabetes Mellites Type II, since 2011	Tradolan 50 mg 1-0-0
Chronic osteoarthritis, left upper ankle	Chronic obstructive pulmonary disease (Gold II-III)	Janumet 50/1000 mg 1-0-1
	Apoplex, 2006	Jardiance 10 mg 1-0-0
left upper ankle joint prosthesis, 2006	Arterial hypertension	Teveten Plus 1/2-0-0
chronic osteoarthritis, right lower ankle joint, 2011	Condition after multiembolic ischemia, September 2013, and insult, 2006	Nomexor 5 mg 1-0-1/2
Subtalar arthrodesis on the right foot, February 2012	Condition after craniocerebral trauma with narrow right parafacial hematoma without RF signs	Lasix 40 mg, when needed
shoulder TEP, October 2018	cavotricuspidal isthmus ablation, January 2017, tachycardic episodes, cardioversion after sedacorone bolus	Sortis 80 mg 0-0-1
	Paroxysmal atrial fibrillation	Lansobene 30 mg 1-0-0
	Condition after appendectomy and tonsillectomy	Magnosolv 1-0-0
	prolonged reversible ischemic neurological deficit with acute aphasia, October 2018	Seebri breezhaler 44 µg, when needed
	Condition after Fracture Costae IIS4-8 right, October 2018	Seretide discus forte 50 µg 1-0-1
	Iron deficiency anemia	Diamicron 30 mg 1-0-0
	Ultra-short Barret esophagus	Seloken
	Large axial hiatal hernia	Eliquis 5 mg 1-0-1
	Steatosis hepatitis	
	Sigmoid diverticulosis	
	Accentuated gall bladder with polyps	
	Non-stenosing coronary stenosis, 2017	
	Pulsarythmias with pulse around 180 beats per minute	
	Known spinal stenosis C3/C4 and C4/C5	
	Hyperlipidemia	
	Anaphylactic reaction after eating pikeperch, celery, carrots, potatoes	
	allergies: penicillin, flavor enhancers, raw fruit and lettuce, fabric softener and starch in laundry	

## Data Availability

Due to the sensitivity of the raw data, additional information can be obtained from the first author.
